# Correlation between salivary alpha-amylase, anxiety, and game records in the archery competition

**DOI:** 10.20463/jenb.2016.0050

**Published:** 2016-12-31

**Authors:** In Soo Lim

**Affiliations:** 1 Department of Physical Education, Changwon National University, Changwon Republic of Korea

**Keywords:** Salivary alpha-amylase, Anxiety, Archery competition

## Abstract

**[Purpose]:**

This study was aimed to investigate the relationship between psychological and physiological changes and performance in archery, which is strongly influenced by psychological factors including concentration, tension, anxiety, and stress.

**[Methods]:**

A total of 19 athletes from women’s colleges who participated in the 30 m individual competition at the 34th President’s Cup National Archery Competition in July 2016 were included in this study. The anxiety levels of the participants were assessed on a 10-point Likert scale, with 1 corresponding to “not at all” and 10 to “extremely anxious.” Saliva samples were collected as follows: 10 min before the game (pre-10), 1 min before the game (pre-1), and 10 min after the game (post-10). Repeated measures general linear model ANOVA was performed to compare the mean values of salivary alpha amylase (sAA) concentrations and anxiety levels. The correlations between sAA, anxiety, and game records were analyzed using the Pearson’s correlation method.

**[Results]:**

The sAA concentrations increased significantly in pre-1 and post-10, but not in pre-10 samples. Moreover, anxiety levels increased in both pre-1 and post-10 samples, but not in pre-10 samples. Anxiety and sAA were positively correlated (p < 0.01), while sAA and game records, or anxiety and game record were negatively correlated (p < 0.01).

**[Conclusion]:**

During the archery competition, the level of cognitive anxiety increased, sAA concentrations increased, and performance decreased. The study findings suggest that during archery competitions, anxiety hinders performance, and this effect may be related to the increase in sAA levels.

## INTRODUCTION

Psychological factors significantly affect the performance during archery compared with other sports. Many skilled archers are not able to perform as well as they usually can owing to tension, anxiety, and stress caused by the actual game^1^. Generally, stress activates the hypothalamic-pituitary-adrenal (HPA) axis and the sympatho-adrenal medullary (SAM) system. The activity of the HPA axis increases cortisol levels while the activity of the SAM system increases catecholamine levels^2^. The measurement of stress levels is generally conducted by measuring blood cortisol and catecholamine levels. In addition, the level of stress can be also measured by simply taking saliva samples from athletes while they compete in sports games^3^. However, it is known that while blood catecholamines do not reflect the concentration of salivary catecholamines, salivary alpha amylase (sAA) reflects blood catecholamines^4-7^. Indeed, sAA secreted into the saliva to digest starch increases when sympathetic nerves are activated during physical and mental stress situations^8,9^. It is also known that sAA increases owing to stress, anxiety, and stressful stimuli^10-12^, and that it responds faster than salivary cortisol^5^.

It has been previously reported that competitive stress caused by Taekwondo competitions increases sAA levels^13-15^. Since sAA is sensitive to psychological stress, it is used as a marker to evaluate anxiety, tension, and stress levels in sports^16,17^. To date, no research has been conducted on psychologically sensitive sports, such as shooting and archery, and field studies investigating a link between the psychological and physiological factors and the performance during actual sporting events are still lacking. Therefore, this study was aimed to investigate the link between psychological and physiological changes and performance in archery, which is strongly influenced by psychological factors, such as concentration, tension, anxiety, and stress. For this purpose, sAA, anxiety, and game records were evaluated in an actual archery competition and the relationships between these parameters were analyzed.

## METHODS

### Participants

This study was conducted with 19 athletes from women’s colleges, who participated in the 30 m individual competition of the 34th President’s Cup National Archery Competition in July 2016. The participants were archers with a career of more than 10 years, who were not taking any substances or medications and did not have any clinical disease, especially intra-oral or inflammatory diseases. The mean age of the archers was 22.12 ± 1.47 y, their mean height was 167.40 ± 5.22 cm, and their mean body weight was 162.07 ± 6.21 kg.

### Assessment of anxiety and game records

The anxiety levels of the participating archers were assessed on a 10-point Likert scale based on the scale introduced Beck et al.^18^, with 1 corresponding to “not at all” and 10 corresponding to “extremely anxious.” With respect to the evaluation method, the cognitive anxiety of the study participants was measured 10 min before playing archery (pre-10), 1 min before playing archery (pre-1), and 10 min after playing archery (post-10). During the 30 m target competition, each archer shot six arrows per end in six ends in total. For the evaluation of the athletes’ performance, the scores of all 36 shots were recorded for each archer.

### Saliva collection and analysis

Participants were instructed to fast for at least 1 h before saliva specimens were collected. Ten minutes prior to the collection of saliva, participants washed their mouth and saliva samples were collected 10 min before the game, 3 min before the game, and 10 min after the game. Briefly, the participants were instructed to chew on an absorbent swab placed in their mouth, after which saliva samples were placed in collection tubes and stored in the refrigerator. The sAA analysis was performed using enzyme-linked immunosorbent assay (ELISA) with the VersaMax™ Microplate Reader (Molecular Devices, Sunnyvale, CA, USA).

### Statistical analysis

The data were analyzed using SPSS software (version 23). The values of all the variables were presented as the mean ± standard deviation (S.D.). Repeated measures general linear model ANOVA was performed to compare the mean values of sAA concentrations and anxiety levels. The correlations between sAA, anxiety, and game records were analyzed using the Pearson’s correlation method. Statistical significance was considered at p ≤ 0.05, except for correction for multiple testing.

## RESULTS

### Anxiety, sAA, and game records

During the actual archery competition, the individual records of the 19 participants were 355, 354, 354, 354, 352, 352, 352, 352, 352, 352, 349, 349, 349, 347, 345, 343, 342, 342, and 341 points, and the average score was 349.20 ± 4.6 points. The anxiety levels of the participants during the competition were also measured and were as follows: pre-10: 5.89 ± 1.8, pre-1: 8.15 ± 1.4, and post-10: 6.76 ± 1.8. The verification test results showed significant differences (F = 7.05, p < 0.05). The pre-1 and post-10 values showed a significant increase in the anxiety level compared with pre-10 values. The changes in the sAA concentrations were 43.80 ± 11.3 U/ml in the pre-10, 87.40 ± 22.4 U/ml in the pre-1, and 78.20 ± 20.4 U/ ml in the post-10. The verification test results showed significant differences (F = 4.30, p < 0.05). The pre-1 and post-10 showed a significant increase in the sAA concentrations compared with pre-10.

**Figure 1.**
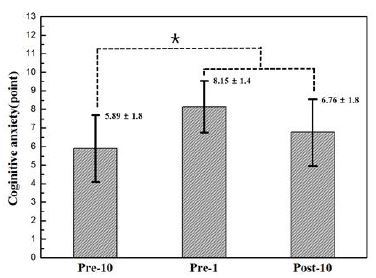
Changes in the cognitive anxiety during pre-10, pre-1, and post-10. pre-10: 10 min before the archery competition; pre-1: 1 min before the archery competition; post-10: 10 min after the archery competition; *: The pre-1 and post-10 showed a significant increase (p<.05) in the cognitive anxiety compared to pre-10.

**Figure 2.**
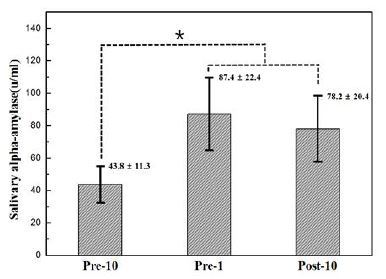
Changes in the sAA level during pre-10, pre-1, and post-10. pre-10: 10 min before the archery competition; pre-1: 1 min before the archery competition; post-10: 10 min after the archery competition; *: The pre-1 and post-10 showed a significant increase (p<.05) in the sAA level compared to pre-10.

### Correlations

The results of the correlations between the sAA, anxiety, and game records in an actual archery competition are summarized in Table 1. There was a significant correlation between all the measurement variables. In other words, the study results showed a high correlation between the sAA concentration and anxiety level, between the sAA concentration and game records, and between the anxiety level and game records.

**Table 1.** Correlation between sAA, anxiety, and game record in archery competition.

	Anxiety	Game record
sAA	.62**^a^	-.87**^b^
Anxiety		-.84**^c^

sAA: salivary alpha amylase

a: significant positive correlation (p < 0.01) between sAA and anxiety

b: significant negative correlation(p < 0.01) between sAA and game record

c: significant negative correlation(p < 0.01) between anxiety and game record

In other words, we observed a positive correlation between anxiety and sAA; as the anxiety level increased, the sAA concentrations increased as well (p < 0.01). However, a negative correlation was observed between anxiety and game records; as the anxiety level increased, performance during the competition decreased (p < 0.01). In addition, game records and sAA also exhibited a negative correlation; as performance decreased, the sAA concentrations increased (p < 0.01).

## DISCUSSION

During the actual archery competition, the individual records of 19 participants showed high scores ranging between 355 and 352 points and medium scores ranging between 349 and 341 points. For the anxiety levels, pre-1 and post-10 values were significantly higher than pre-10 values. Interestingly, anxiety levels tended to be maintained even after the end of the game, and the anxiety levels of the athletes with middle scores were significantly higher than those of athletes achieving high scores. This result indicates that the level of cognitive anxiety directly affected the performance. In a related study, it has been found that as the athletes were under higher pressure during a competition, performance decreased^10,12^. Parnabas et al.^1^ analyzed the levels of cognitive anxiety of 106 athletes and reported a strong negative correlation between athletic performance and anxiety.

Previously, it has been reported that high sAA levels were found in patients with chronic stress^10,19^, and acute stress resulted in elevated sAA levels^20,21^. Nater et al.^10^ reported that when the scenes of corneal transplantation surgery were shown to adults as a stimulus source of stress, sAA concentrations were observed to increase rapidly. In line with these previous findings, we also found that archery competition increased the level of anxiety and sAA concentrations. In other words, a significant increase in sAA levels was found in the pre-3 and post-10 values compared with the pre-10 values in an archery competition. These results may suggest that stress activates the HPA axis and SAM system, thereby increasing blood catecholamine concentrations, which in turn is reflected by sAA concentrations. In this study, the increased sAA concentrations were maintained for a while after the game ended. Moreover, these levels were significantly higher in participants with middle scores than in participants with high scores. These results indicate that as the level of sAA increased, the performance decreased. In a related study, Edmonds et al.^22^ reported that swimmers showed an increase in sAA levels during the international swimming meet season. Capranica et al.^15^ reported that sAA increased as the competitive stress caused by the Taekwondo competition increased. Therefore, sAA can be used as a parameter to evaluate anxiety in actual sports events, and as a marker for evaluating athletic performance. Thus, it is suggested that anxiety decreases athletic performance in sportive competitions and that this association between anxiety and performance is related to an increase in sAA concentrations. These results suggest that in order to improve athletic performances, it may help to organize a training program taking into account the changes in sAA concentrations during actual sports competitions of archery or shooting.

In this study, sAA, anxiety, and game records were analyzed and evaluated in an archery competition where athletic performance was greatly affected by psychological factors, such as concentration, tension, anxiety, and stress. The study results showed that as the level of cognitive anxiety increased during the archery competition, performance decreased and the sAA concentrations became elevated. In sports competitions, anxiety lowers performance and this study suggests that this correlation is related to the increase in sAA levels. In sports competitions, the level of sAA can be used as an effective marker for assessing the anxiety level and performance.
